# Satellite DNA at the Centromere Is Dispensable for Segregation Fidelity

**DOI:** 10.3390/genes10060469

**Published:** 2019-06-20

**Authors:** Annalisa Roberti, Mirella Bensi, Alice Mazzagatti, Francesca M. Piras, Solomon G. Nergadze, Elena Giulotto, Elena Raimondi

**Affiliations:** Department of Biology and Biotechnology “L. Spallanzani”, University of Pavia, Via Ferrata 1, 27100 Pavia, Italy; annalisamanuel.roberti01@universitadipavia.it (A.R.); mirella.bensi@unipv.it (M.B.); a.mazzagatti@qmul.ac.uk (A.M.); mfrancesca.piras@unipv.it (F.M.P.); solomon.nergadze@unipv.it (S.G.N.)

**Keywords:** centromere, horse, satellite DNA, satellite-free centromere, segregation fidelity, aneuploidy, micronucleus assay, Fluorescence In Situ Hybridization

## Abstract

The typical vertebrate centromeres contain long stretches of highly repeated DNA sequences (satellite DNA). We previously demonstrated that the karyotypes of the species belonging to the genus *Equus* are characterized by the presence of satellite-free and satellite-based centromeres and represent a unique biological model for the study of centromere organization and behavior. Using horse primary fibroblasts cultured in vitro, we compared the segregation fidelity of chromosome 11, whose centromere is satellite-free, with that of chromosome 13, which has similar size and a centromere containing long stretches of satellite DNA. The mitotic stability of the two chromosomes was compared under normal conditions and under mitotic stress induced by the spindle inhibitor, nocodazole. Two independent molecular-cytogenetic approaches were used—the interphase aneuploidy analysis and the cytokinesis-block micronucleus assay. Both assays were coupled to fluorescence in situ hybridization with chromosome specific probes in order to identify chromosome 11 and chromosome 13, respectively. In addition, we tested if the lack of centromeric satellite DNA affected chromatid cohesion under normal and stress conditions. We demonstrated that, in our system, the segregation fidelity of a chromosome is not influenced by the presence of long stretches of tandem repeats at its centromere. To our knowledge, the present study is the first analysis of the mitotic behavior of a natural satellite-free centromere.

## 1. Introduction

The centromere is a *locus* essential for chromosome segregation and genome integrity; this function is carried out by supporting kinetochore assembly and attachment to spindle microtubules. Most vertebrate centromeres are composed of long stretches of highly repeated DNA sequences named satellite DNA [1,2]. Nonetheless, kinetochore assembly is not dependent on the primary DNA sequence, and the centromeres are epigenetically determined, the modified H3 histone CENP-A (CENtromere Protein A) being the marker of centromere function. The existence of completely satellite-free pathological [3,4,5] and natural [6,7,8,9,10] centromeres raises the question of whether satellite DNA at centromeres plays any functional role. Centromeric repetitive DNA is typically devoid of active genes, thus it may aid the formation of a heterochromatic environment, which would favor the stability of the chromosome during mitosis and meiosis [1,2,5]. In several species, centromeric satellite DNA is transcribed, and it has been suggested that transcription of the centromeric regions may be important for chromatin opening and CENP-A loading; these transcripts are believed to provide a flexible scaffold that allows assembly or stabilization of the kinetochore proteins and may act in trans on all or on a subset of chromosomes independently of the primary DNA sequence [11,12,13,14]. Moreover, most mammalian centromeric satellite DNA sequences contain binding sites for the constitutive centromeric protein CENP-B (CENtromere Protein B), whose role is still a matter of debate [15,16,17,18]. 

A crucial issue in centromere biology concerns the contribution of satellite DNA to chromosome segregation fidelity. To our knowledge, the mitotic stability of satellite-free centromeres was not carefully investigated. Data from the analysis of pathologic satellite-free centromeres indicate that these marker chromosomes are often present in the individual in mosaic form. Since most human neocentromeres give rise to partial trisomy or tetrasomy, it is plausible to suppose that the selective disadvantage of partial aneuploidy is responsible for this mosaicism [5].

Human artificial chromosomes containing fully functional centromeres have been constructed by different approaches [19,20,21,22], and it was demonstrated that to be propagated in culture, they require alpha-satellite arrays, including binding sites for the CENP-B protein (CENP-B box) [23,24]. Later on, human artificial chromosomes with a conditional centromere were used to manipulate the epigenetic state of chromatin and to elucidate the requirements for proper centromere function [25,26,27]. These studies together with high resolution immunofluorescence on chromatin fibers [28] revealed that a centromere-specific balance between typical euchromatic and typical heterochromatic post-translational histone modifications is essential for kinetochore activity, with highly repetitive DNA stretches having a central role.

We previously described a biological model system represented by species belonging to the genus *Equus* that is especially suitable for the dissection of centromere function [6,14,29,30,31,32,33,34]; in these species, the centromere function and the position of satellite DNA turned out to be often uncoupled [30]. Moreover, centromere repositioning (that is, centromere movement along the chromosome without rearrangement [35]) was unexpectedly frequent in *Equus* species [36] and generated satellite-free centromeres. In particular, one horse (ECA, *Equus Caballus*) chromosome (ECA11) and 16 donkey (EAS, *Equus Asinus*) chromosomes (EAS4, 5, 7, 8, 9, 10, 11, 12, 13, 14, 16, 18, 19, 27, 30, X) were demonstrated to be completely satellite-free at the sequence level [6,34]. 

Strikingly, the satellite-free centromere of horse chromosome 11 and the 16 satellite-free donkey centromeres showed a sliding behavior. In other words, we demonstrated that the functional centromeric domain as defined by CENP-A binding at these centromeres can move within a region spanning 500-600 kb, thus generating functional alleles or “epialleles” [33,34]. 

In the present paper, we analyzed the mitotic stability of horse chromosome 11 (ECA11), whose centromere is completely satellite-free, and compared it with that of chromosome 13 (ECA13), which has similar size and a centromere containing long stretches of the canonical horse centromeric satellite DNA families [30,32]. The comparison was performed under normal conditions and after exposure of the cells to mitotic stress induced by the spindle inhibitor nocodazole. Two chromosome stability assays, interphase aneuploidy analysis and the cytokinesis-block micronucleus assay, were combined with fluorescence in situ hybridization (FISH) with chromosome specific probes. 

## 2. Materials and Methods 

### 2.1. Cell Line and Metaphase Spread Preparation 

A horse skin fibroblast cell line was previously established [30]. The fibroblasts were cultured in Dulbecco’s modified Eagle’s medium (CELBIO) supplemented with 20% fetal calf serum (Bio-west), 2 mM glutamine, 2% non-essential amino acids, and 1x penicillin/streptomycin. Cells were maintained at 37 °C in a humidified atmosphere of 5% CO2.

Mitotically active cells were collected by flushing the medium on the cell monolayer. Metaphase spreads were prepared following the standard air-drying procedure.

### 2.2. Drug Treatment and Cytokinesis-Blok

Fifty thousand cells were seeded in 5 cm diameter Petri dishes containing 24 × 24 mm coverslips. After a 24 h culture period, cells were exposed to nocodazole (Sigma, St. Louis, MO, USA) 100 nM. Since nocodazole is soluble in DiMethyl SulfOxide (DMSO), control cells were treated with the same DMSO concentration used for nocodazole treatment. For the cytokinesis-block micronucleus (CBMN) assay, 24 h after the addition of nocodazole (or DMSO in control samples) cytochalasin-B (Cyt-B), 5 μg/mL was added to the cultures. After 48 h of nocodazole treatment, the slides were processed for interphase nuclei and micronuclei preparations as follows. Slides were treated with 75 mM KCl hypotonic solution and incubated at 37 °C for 15 min. Cold (−20 °C) fixative (methanol:acetic acid—3:1) was added for 30 min. The fixation was repeated twice. 

### 2.3. BAC Extraction and FISH

Two bacterial artificial chromosomes (BACs) derived from horse CHORI-241 BAC library were used (CHORI241-21D14, chr11: 27,639,936-27,829,952; CHORI241-22C1, chr13: 7,346,775-7,544,907). The BACs were extracted from 100 mL of liquid culture (LB medium and chloramphenicol 12.5 μg/mL). The extraction was carried out with Qiagen Plasmid purification kit®, according to the supplier instructions. 

The BACs were labeled by nick translation with Cy3-dUTP (Cyanine 3-dUTP, Perkin Elmer) and hybridized to metaphase spreads, interphase nuclei, or micronuclei preparations. For each slide, 250 ng of probe were used. Slides were denatured at 72 °C in 70% formamide 2 × SSC and immediately incubated overnight at 37 °C with the denatured probe in 50% formamide 2 × SSC. Post-hybridization washes were performed in 50% formamide 2 × SSC at 42 °C. Slides were counterstained with DAPI (4′, 6′-Diamidino-2-phenylindole hydrochloride, 1 μg/mL) and mounted in DAKO mounting medium. 

A fluorescence microscope (Zeiss Axioplan) equipped with a cooled, grey scale charge coupled device (CCD) camera (Photometrics) was used for image capturing. Digital grey scale images for Cy3 and DAPI fluorescence signals were acquired separately, pseudo-colored, and merged using the IpLab software. 

## 3. Results and Discussion

### 3.1. Interphase Aneuploidy Analysis

Aneuploidy is the presence of extra or missing chromosomes in a cell. Different mechanisms can lead to aneuploidy: (i) nondisjunction, which can involve single or multiple chromosomes; (ii) merotelic attachment, which occurs when one kinetochore is attached to both mitotic spindle poles; and (iii) formation of multipolar/monopolar spindles. FISH in interphase is a rapid and reliable molecular cytogenetic approach for the targeted detection of aneuploidy [37]. The number of FISH signals per cell is counted using chromosome specific probes. This analysis is currently used for human prenatal diagnosis and for the rapid screening of cancer cell populations. 

**Figure 1:**
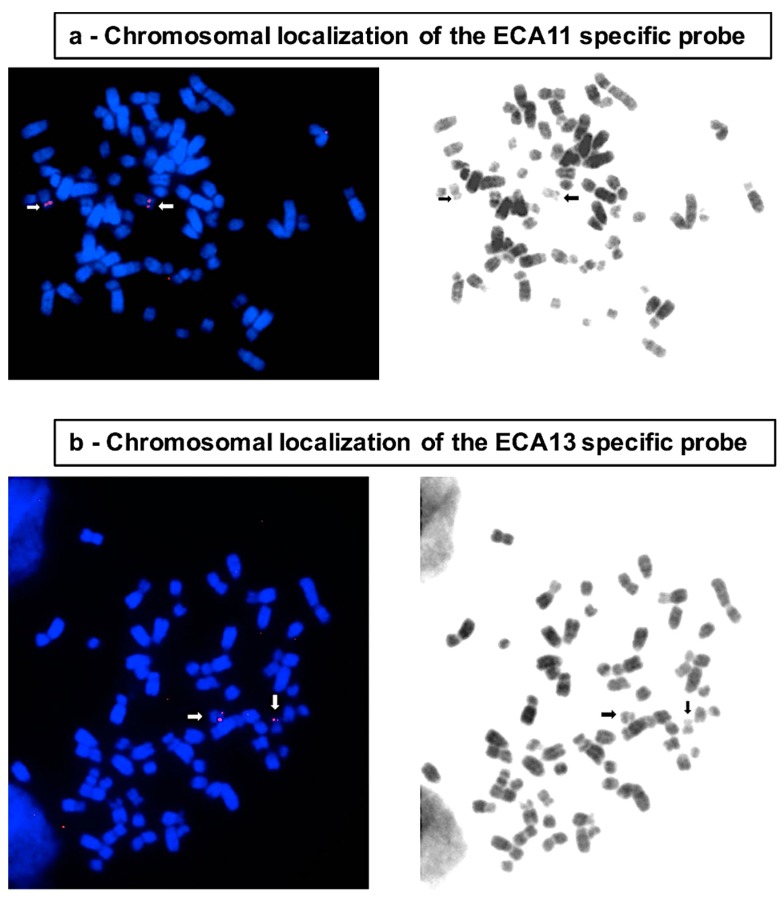
Chromosomal localization by fluorescence in situ hybridization (FISH) of the bacterial artificial chromosome (BAC) probes used. (**a**) FISH with the ECA11 specific BAC probe. Chromosome 11 is marked by the arrows. (**b**) FISH with the ECA13 specific BAC probe. Chromosome 13 is marked by the arrows. Probe localization is marked by the red FISH signals in the images on the left. Chromosomes are counterstained with 4′, 6′-Diamidino-2-phenylindole hydrochloride (DAPI). Reverse DAPI banding is shown in the images on the right.

In the present study, we used this test to compare the segregation fidelity of horse chromosome 11, whose centromere is satellite-free, with that of chromosome 13, which has a similar size and the centromeric function associated to satellite DNA [30]. Interphase FISH experiments were carried out with two BAC probes derived from the horse CHORI-241 BAC library [38] and specific for ECA11 and ECA13, respectively (CHORI241-21D14, chr11: 27,639,936–27,829,952 and CHORI241-22C1, chr13: 7,346,775–7,544,907). The localization of the ECA11 centromeric BAC is shown in Figure 1a. Since the centromere of chromosome 13 contains long stretches of satellite DNA and no single copy sequence co-localizes with the centromere, for the identification of this chromosome, we used a BAC clone mapping on the short arm close to the centromere (Figure 1b).

**Figure 2:**
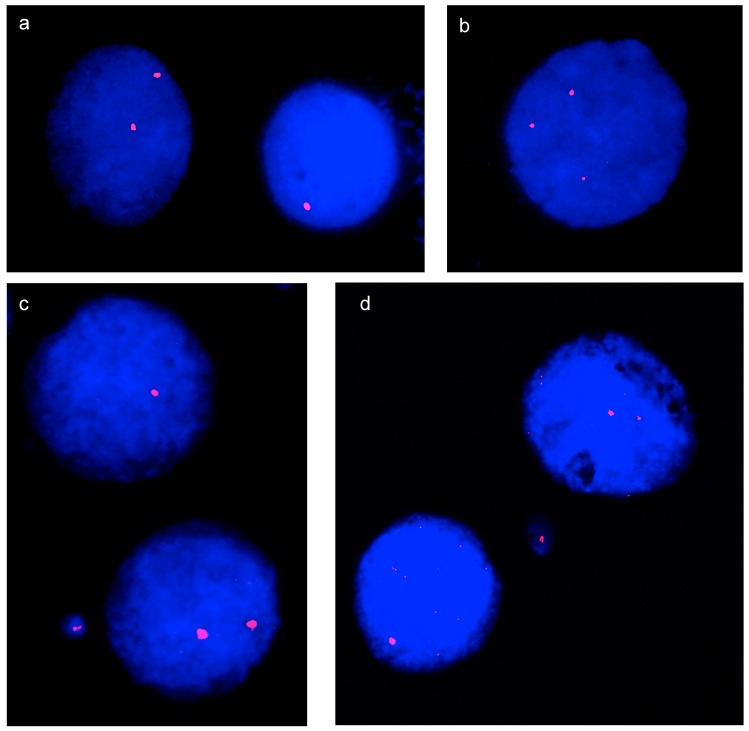
FISH on interphase nuclei and on micronuclei. (**a**) A nucleus, disomic for chromosome 11, is flanked by a monosomic one. (**b**) Nucleus trisomic for chromosome 13. (**c**) Bi-nucleated Cyt-B treated cell with a disomic nucleus, a monosomic nucleus, and a chromosome 11 positive micronucleus. (**d**) Bi-nucleated Cyt-B treated cell with a disomic nucleus, a monosomic nucleus, and a chromosome 11 positive micronucleus.

To evaluate the frequency of aneuploid cells, the number of fluorescence spots corresponding to each probe was counted by visual inspection on interphase nuclei; numbers differing from two indicated an aneuploid condition. Since the frequency of chromosome specific aneuploidy and micronuclei was expected to be low, even minor differences in the efficiency or the stability of different fluorophores would impair the results. Therefore, to minimize experimental error, separate FISH experiments using the same fluorophore (Cy3) were carried out with probes for chromosome 11 and chromosome 13. Figure 2 shows examples of the results of FISH experiments. The number of signals was then counted on interphase nuclei hybridized with the chromosome 11 or with the chromosome 13 specific probe. 

Samples of 4000 interphase nuclei from three replicate experiments for chromosome 11 and 13, respectively, were analyzed under control conditions. The results of the analysis are reported in Table 1. Statistical analysis of the difference in the number of nuclei aneuploid for chromosome 11 or 13 was performed with the χ^2^ test. The mitotic behavior of the two chromosomes was comparable, because the difference between the number of cells aneuploid for chromosome 11 (1,90) and for chromosome 13 (1,98) was not statistically significant, and the standard error of the replicates is reported in the table. Table 1 also shows that the percentages of nullisomic, monosomic, and trisomic nuclei for the two chromosomes were similar. A few nuclei with four signals were observed: 0.40% and 0.36% for chromosome 11 and for chromosome 13, respectively. However, since this assay does not allow determination of whether these nuclei correspond to tetrasomic or tetraploid conditions, they were not included in the analysis of aneuploid cells.

Horse fibroblasts were then treated with 100 nM nocodazole for 48 h, and the number of aneuploid nuclei was counted in samples of about 1000 nuclei from two replicates for chromosomes 11 and 13, respectively, under control and stress conditions (Table 2). Nocodazole is a well described antimitotic agent that interferes with the function of spindle and cytoplasmic microtubules by binding to tubulin. The use of this drug was aimed at amplifying the difference, if any, in segregation fidelity between the satellite-free ECA11 centromere and the satellite-based ECA13 centromere and also at identifying possible differences in their sensitivity to conditions perturbing cell division.

A highly significant increase in the number of aneuploid nuclei was observed for both chromosomes after drug treatment (χ^2^ test, p-value 3 × 10^−3^ and 1 × 10^−4^, respectively). However, the difference in the number of nuclei aneuploid for chromosomes 11 or 13, as assayed by the χ^2^ test, was not significant either under control conditions or after drug treatment. This result indicates that satellite-free and satellite based horse centromeres are equally sensitive to this antimitotic drug.

### 3.2. Micronucleus Assay and Cytokinesis-Block MicroNucleus (CBMN) Assay 

The micronucleus assay is a mutagenic test for the detection of small membrane-bound DNA fragments (i.e., micronuclei in the cytoplasm of interphase cells) [39,40]. Centric and acentric chromosome fragments as well as whole chromosomes unable to migrate to one pole during anaphase can be included into micronuclei. Two mechanisms, chromosome breakage and disturbance of chromosome segregation, may lead to the formation of micronuclei. 

To test whether the formation of micronuclei is influenced by the presence/absence of centromeric satellite DNA, we performed the micronucleus assay using the chromosome specific BAC probes described above. Also, in this assay, to minimize the experimental error, micronuclei containing chromosome 11 or chromosome 13 were counted in independent experiments in which both probes were red labeled with Cy3. 

In the same samples of 4000 nuclei previously analyzed for aneuploidy, we calculated the frequency of spontaneously occurring micronuclei. The results are reported in Table 3. The frequencies of spontaneously occurring micronuclei (1.6 and 1.4%) were comparable to those previously observed in human primary fibroblasts [41,42], and no significant difference in the number of chromosome 11 or chromosome 13 containing micronuclei was observed, as determined by the Fisher’s exact test. The standard error calculated in the three replicates is reported in the table.

As for the aneuploidy assay, we then analysed micronuclei in cells treated with nocodazole. To make sure that the cells being scored have completed mitosis during nocodazole treatment we performed the Cytokinesis-Block MicroNucleus (CBMN) assay [43,44]. In this assay scoring is specifically restricted to once-divided bi-nucleated (BN) cells, after blocking cytokinesis with cytochalasin-B (Cyt-B), an inhibitor of microfilament ring assembly required for the completion of cytokinesis. 

Cells were treated with 100 nM nocodazole for 48 h. During the last 24 h Cyt-B was added to the cultures. Two replicates were performed for each condition and in each experiment a total of 2.000 BN cells was scored. The results of the CBMN assay are reported in Table 4. 

After nocodazole exposure, a highly significant increase in the total number of micronuclei was observed (χ^2^ test, *p*-value 3 × 10^−5^ and 9 × 10^−4^, respectively), thus demonstrating the effectiveness of the treatment. When the numbers of chromosome 11 and chromosome 13 containing micronuclei were compared, no difference between the two chromosomes was observed under control conditions or after exposure to nocodazole (due to the small number of chromosome positive micronuclei observed, the Fisher’s exact test was used in this case). Figure 2c,d show examples of bi-nucleated cells containing micronuclei positive for chromosome 11 and chromosome 13, respectively. 

The results of the micronucleus assay confirmed that the mitotic behavior of the two chromosomes was comparable and that the absence of satellite DNA at the centromere did not affect its sensitivity to the spindle inhibitor nocodazole. These results are in agreement with those obtained by interphase nuclei analysis. Thus, two independent molecular-cytogenetic approaches demonstrated that, in the horse fibroblast system, segregation fidelity is not influenced by the presence of satellite DNA sequences at the centromere.

### 3.3. Chromatid Cohesion Analysis

Literature data report that aurora B-kinase, which regulates chromosome-spindle attachments, mislocalizes at pathological human satellite-free centromeres [45,46]. Aurora B is part of a protein network, the chromosomal passenger complex (CPC), which regulates proper chromatid segregation. In the hypothesis that, in the horse system, the absence of satellite DNA at the centromere could affect CPC function, thus perturbing chromatid cohesion during cell division, we analyzed samples of 200 metaphase spreads from control fibroblast cultures and from cultures treated with 100 nM nocodazole. Again, FISH with the chromosome 11 and the chromosome 13 probes was performed in order to compare the behavior of the two centromeres. We never observed chromatid cohesion defects for any chromosome in either control samples or under mitotic stress conditions.

## 4. Conclusions

The species belonging to the genus *Equus* are characterized by karyotypes where chromosomes with canonical satellite-based centromeres are present together with chromosomes with satellite-free centromeres [6,14,29,30,31,32,33,34]. This exceptional biological model offers the opportunity to directly investigate, in a natural environment, the behavior of these differently organized centromeres. 

Here, we analyzed the mitotic behavior of the satellite-free centromere of ECA11 and compared it with that of the canonical, satellite-based, ECA13 centromere. Our results demonstrated that the segregation accuracy of these two chromosomes is similar, thus suggesting that satellite DNA is dispensable for transmission fidelity.

Sequence analysis of the centromere of horse chromosome 11 showed that no motif resembling a CENP-B box is present (unpublished results). Consequently, our results suggest that, in the horse system, the CENP-B protein does not play a central role in chromosome segregation.

The function of satellite DNA at the centromere is a matter of debate, literature data suggesting that centromeric and/or pericentromeric repeated DNA sequences create the chromatin environment needed for sister chromatid cohesion and for kinetochore recruitment [5,12,13,47]. Indeed, the large majority of vertebrate centromeres contain highly repeated DNA sequences [1,2]. The widespread presence of repeated DNA at natural centromeres suggests that there is a positive selection for this kind of arrangement. The results presented here demonstrate that, at least in the horse, the absence of satellite DNA at the centromere does not affect chromosome segregation. Therefore, we postulate that satellite DNA may play roles other than the maintenance of segregation fidelity. One possibility based on our previous results on centromere sliding [33,34] is that satellite DNA may contribute to constrain the borders of the functional centromeric domain within non-coding genomic regions.

## Figures and Tables

**Table 1:** Interphase FISH under normal conditions.

Chromosome	Total Number of Nuclei	Diploid Nuclei (%)	Aneuploid Nuclei (% ± SE)	Type of Aneuploidy	Number (%)
*ECA11*	4000	3924 (98,10)	76 (1.90 ± 0.196)	*nullisomy*	5 (0,12)
				*monosomy*	30 (0,75)
				*trisomy*	41 (1,02)
*ECA13*	4000	3921 (98,03)	79 (1.98 ± 0.329)	*nullisomy*	8 (0,20)
				*monosomy*	32 (0,80)
				*trisomy*	39 (0,98)

**Table 2:** Interphase FISH under mitotic stress.

Chromosome	Total Number of Nuclei	Diploid Nuclei (%)	Aneuploid Nuclei (% ± SE)	Type of Aneuploidy	Number (%)
**Control**					
*ECA11*	1107	1084 (97,92)	23 (2.08 ± 0.014)	*nullisomy*	0
				*monosomy*	11 (0,99)
				*trisomy*	12 (1,08)
*ECA13*	1117	1098 (98,30)	19 (1.70 ± 0.057)	*nullisomy*	0
				*monosomy*	11 (0,98)
				*trisomy*	8 (0,72)
**Nocodazole 100 nM**					
*ECA11*	1045	1000 (95,70)	45 (4.31 ± 0.085)	*nullisomy*	0
				*monosomy*	13 (1,24)
				*trisomy*	32 (3,01)
*ECA13*	1023	976 (95,40)	47 (4.60 ± 0.042)	*nullisomy*	0
				*monosomy*	12 (1,17)
				*trisomy*	35 (3,42)

**Table 3:** Spontaneously occurring micronuclei under control conditions without cytokinesis-block.

Chromosome	Total Number of Nuclei	Total Number of Micronuclei (% ± SE)	Micronuclei Containing the Chromosome (% ± SE)	Micronuclei not Containing the Chromosome (%)
*ECA11*	4000	63 (1.6 ± 0.098)	7 (11.1 ± 1.143)	56 (88,9)
*ECA13*	4000	55 (1.4 ± 0.127)	6 (10.9 ± 2.390)	49 (89,1)

**Table 4:** Cytokinesis-Block MicroNucleus assay under mitotic stress.

Chromosome	Total Number of BN Cells	Total Number of Micronuclei (% ± SE)	Micronuclei Containing the Chromosome (% ± SE)	Micronuclei not Containing the Chromosome (%)
**Control**				
*ECA11*	2000	31 (1.6 ± 0.002)	6 (19.4 ± 0.019)	25 (80,6)
*ECA13*	2000	32 (1.6 ± 0.000)	6 (18.8 ± 0.000)	26 (81,2)
**Nocodazole 100 nM**				
*ECA11*	2000	75 (3.8 ± 0.004)	11 (14.7 ± 0.0004)	64 (85,3)
*ECA13*	2000	65 (3.3 ± 0.009)	10 (15.4 ± 0.0180)	55 (84,6)

## References

[1] Plohl M., Luchetti A., Mestrovic N., Mantovani B. (2008). Satellite DNAs between selfishness and functionality: Structure, genomics and evolution of tandem repeats in centromeric (hetero) chromatin. Gene.

[2] Plohl M., Mestrovic N., Mravinac B. (2014). Centromere identity from the DNA point of view. Chromosoma.

[3] Voullaire L.E., Slater H.R., Petrovic V., Choo K.H. (1993). A functional marker centromere with no detectable alpha-satellite, satellite III, or CENP-B protein: Activation of a latent centromere?. Am. J. Hum. Genet..

[4] Choo K.H. (1997). Centromere DNA dynamics: Latent centromeres and neocentromere formation. Am. J. Hum. Genet..

[5] Marshall O.J., Chueh A.C., Wong L.H., Choo K.H. (2008). Neocentromeres: New insights into centromere structure, disease development, and karyotype evolution. Am. J. Hum. Genet..

[6] Wade C.M., Giulotto E., Sigurdsson S., Zoli M., Gnerre S., Imsland F., Lear T.L., Adelson D.L., Bailey E., Bellone R.R. (2009). Genome sequence, comparative analysis, and population genetics of the domestic horse. Science.

[7] Shang W.H., Hori T., Toyoda A., Kato J., Popendorf K., Sakakibara Y., Fujiyama A., Fukagawa T. (2010). Chickens possess centromeres with both extended tandem repeats and short non-tandem-repetitive sequences. Genome Res..

[8] Locke D.P., Hillier L.W., Warren W.C., Worley K.C., Nazareth L.V., Muzny D.M., Yang S.P., Wang Z., Chinwalla A.T., Minx P. (2011). Comparative and demographic analysis of orang-utan genomes. Nature.

[9] Gong Z., Wu Y., Koblizkova A., Torres G.A., Wang K., Iovene M., Neumann P., Zhang W., Novak P., Buell C.R. (2012). Repeatless and repeat-based centromeres in potato: Implications for centromere evolution. Plant Cell.

[10] Giulotto E., Raimondi E., Sullivan K.F. (2017). The Unique DNA Sequences Underlying Equine Centromeres. Prog. Mol. Subcell. Biol..

[11] Quenet D., Dalal Y. (2014). A long non-coding RNA is required for targeting centromeric protein A to the human centromere. Elife.

[12] Rosic S., Kohler F., Erhardt S. (2014). Repetitive centromeric satellite RNA is essential for kinetochore formation and cell division. J. Cell Biol..

[13] Biscotti M.A., Canapa A., Forconi M., Olmo E., Barucca M. (2015). Transcription of tandemly repetitive DNA: Functional roles. Chromosome Res..

[14] Cerutti F., Gamba R., Mazzagatti A., Piras F.M., Cappelletti E., Belloni E., Nergadze S.G., Raimondi E., Giulotto E. (2016). The major horse satellite DNA family is associated with centromere competence. Mol. Cytogenet..

[15] Masumoto H., Masukata H., Muro Y., Nozaki N., Okazaki T. (1989). A human centromere antigen (CENP-B) interacts with a short specific sequence in alphoid DNA, a human centromeric satellite. J. Cell Biol..

[16] Miga K.H., Newton Y., Jain M., Altemose N., Willard H.F., Kent W.J. (2014). Centromere reference models for human chromosomes X and Y satellite arrays. Genome Res..

[17] Fachinetti D., Han J.S., McMahon M.A., Ly P., Abdullah A., Wong A.J., Cleveland D.W. (2015). DNA Sequence-Specific Binding of CENP-B Enhances the Fidelity of Human Centromere Function. Dev. Cell.

[18] Drinnenberg I.A., Henikoff S., Malik H.S. (2016). Evolutionary Turnover of Kinetochore Proteins: A Ship of Theseus?. Trends Cell. Biol..

[19] Farr C.J., Bayne R.A., Kipling D., Mills W., Critcher R., Cooke H.J. (1995). Generation of a human X-derived minichromosome using telomere-associated chromosome fragmentation. Embo J..

[20] Heller R., Brown K.E., Burgtorf C., Brown W.R. (1996). Mini-chromosomes derived from the human Y chromosome by telomere directed chromosome breakage. Proc. Natl. Acad. Sci. USA.

[21] Raimondi E., Balzaretti M., Moralli D., Vagnarelli P., Tredici F., Bensi M., De Carli L. (1996). Gene targeting to the centromeric DNA of a human minichromosome. Hum. Gene Ther..

[22] Harrington J.J., Van Bokkelen G., Mays R.W., Gustashaw K., Willard H.F. (1997). Formation of de novo centromeres and construction of first-generation human artificial microchromosomes. Nat. Genet..

[23] Masumoto H., Nakano M., Ohzeki J. (2004). The role of CENP-B and alpha-satellite DNA: De novo assembly and epigenetic maintenance of human centromeres. Chromosome Res..

[24] Henikoff J.G., Thakur J., Kasinathan S., Henikoff S. (2015). A unique chromatin complex occupies young alpha-satellite arrays of human centromeres. Sci. Adv..

[25] Nakano M., Cardinale S., Noskov V.N., Gassmann R., Vagnarelli P., Kandels-Lewis S., Larionov V., Earnshaw W.C., Masumoto H. (2008). Inactivation of a human kinetochore by specific targeting of chromatin modifiers. Dev. Cell.

[26] Ohzeki J., Bergmann J.H., Kouprina N., Noskov V.N., Nakano M., Kimura H., Earnshaw W.C., Larionov V., Masumoto H. (2012). Breaking the HAC Barrier: Histone H3K9 acetyl/methyl balance regulates CENP-A assembly. Embo J..

[27] Molina O., Vargiu G., Abad M.A., Zhiteneva A., Jeyaprakash A.A., Masumoto H., Kouprina N., Larionov V., Earnshaw W.C. (2016). Epigenetic engineering reveals a balance between histone modifications and transcription in kinetochore maintenance. Nat. Commun..

[28] Sullivan B.A., Karpen G.H. (2004). Centromeric chromatin exhibits a histone modification pattern that is distinct from both euchromatin and heterochromatin. Nat. Struct. Mol. Biol..

[29] Piras F.M., Nergadze S.G., Poletto V., Cerutti F., Ryder O.A., Leeb T., Raimondi E., Giulotto E. (2009). Phylogeny of horse chromosome 5q in the genus Equus and centromere repositioning. Cytogenet. Genome Res..

[30] Piras F.M., Nergadze S.G., Magnani E., Bertoni L., Attolini C., Khoriauli L., Raimondi E., Giulotto E. (2010). Uncoupling of satellite DNA and centromeric function in the genus Equus. PLoS Genet..

[31] Raimondi E., Piras F.M., Nergadze S.G., Di Meo G.P., Ruiz-Herrera A., Ponsa M., Ianuzzi L., Giulotto E. (2011). Polymorphic organization of constitutive heterochromatin in Equus asinus (2n = 62) chromosome 1. Hereditas.

[32] Nergadze S.G., Belloni E., Piras F.M., Khoriauli L., Mazzagatti A., Vella F., Bensi M., Vitelli V., Giulotto E., Raimondi E. (2014). Discovery and comparative analysis of a novel satellite, EC137, in horses and other equids. Cytogenet. Genome Res..

[33] Purgato S., Belloni E., Piras F.M., Zoli M., Badiale C., Cerutti F., Mazzagatti A., Perini G., Della Valle G., Nergadze S.G. (2015). Centromere sliding on a mammalian chromosome. Chromosoma.

[34] Nergadze S.G., Piras F.M., Gamba R., Corbo M., Cerutti F., McCarter J.G.W., Cappelletti E., Gozzo F., Harman R.M., Antczak D.F. (2018). Birth, evolution, and transmission of satellite-free mammalian centromeric domains. Genome Res..

[35] Montefalcone G., Tempesta S., Rocchi M., Archidiacono N. (1999). Centromere repositioning. Genome Res..

[36] Carbone L., Nergadze S.G., Magnani E., Misceo D., Francesca Cardone M., Roberto R., Bertoni L., Attolini C., Francesca Piras M., de Jong P. (2006). Evolutionary movement of centromeres in horse, donkey, and zebra. Genomics.

[37] Faas B.H., Cirigliano V., Bui T.H. (2011). Rapid methods for targeted prenatal diagnosis of common chromosome aneuploidies. Semin. Fetal Neonatal Med..

[38] Leeb T., Vogl C., Zhu B., de Jong P.J., Binns M.M., Chowdhary B.P., Scharfe M., Jarek M., Nordsiek G., Schrader F. (2006). A human-horse comparative map based on equine BAC end sequences. Genomics.

[39] Kirsch-Volders M., Elhajouji A., Cundari E., Van Hummelen P. (1997). The in vitro micronucleus test: A multi-endpoint assay to detect simultaneously mitotic delay, apoptosis, chromosome breakage, chromosome loss and non-disjunction. Mutat. Res..

[40] Fenech M. (2000). The in vitro micronucleus technique. Mutat. Res..

[41] Rudd N.L., Hoar D.I., Williams S.E., Hennig U.G. (1989). Genotype and the cryopreservation process affect the levels of aneuploidy and chromosome breakage in cultured human fibroblasts. Genome.

[42] Schmidt-Preuss U., Roser M., Weichenthal M., Rudiger H.W. (1990). Elevated frequencies of micronuclei in cultured fibroblasts after freezing and thawing. Mutat. Res..

[43] Kirsch-Volders M., Sofuni T., Aardema M., Albertini S., Eastmond D., Fenech M., Ishidate M., Kirchner S., Lorge E., Morita T. (2003). Report from the in vitro micronucleus assay working group. Mutat. Res..

[44] Fenech M. (2007). Cytokinesis-block micronucleus cytome assay. Nat. Protoc..

[45] Liu D., Vader G., Vromans M.J., Lampson M.A., Lens S.M. (2009). Sensing chromosome bi-orientation by spatial separation of aurora B kinase from kinetochore substrates. Science.

[46] Bassett E.A., Wood S., Salimian K.J., Ajith S., Foltz D.R., Black B.E. (2010). Epigenetic centromere specification directs aurora B accumulation but is insufficient to efficiently correct mitotic errors. J. Cell Biol..

[47] Shang W.H., Hori T., Martins N.M., Toyoda A., Misu S., Monma N., Hiratani I., Maeshima K., Ikeo K., Fujiyama A. (2013). Chromosome engineering allows the efficient isolation of vertebrate neocentromeres. Dev. Cell.

